# First person – Guang Yang

**DOI:** 10.1242/bio.062706

**Published:** 2026-06-01

## Abstract

First Person is a series of interviews with the first authors of a selection of papers published in Biology Open, helping researchers promote themselves alongside their papers. Guang Yang is first author on ‘ Profiling cell proliferation after whole-genome duplication in human cells’, published in BiO. Guang conducted the research described in this article while a PhD student in Ryota Uehara's lab at the Faculty of Advanced Life Science, Hokkaido University, Japan. Guang is now a postdoc in the lab of Tomonori Matsumoto at the Graduate School of Frontier Biosciences, The University of Osaka, Japan, investigating cell fate decision, physiological and pathological contribution of the whole-genome duplication cell.

**Figure:**
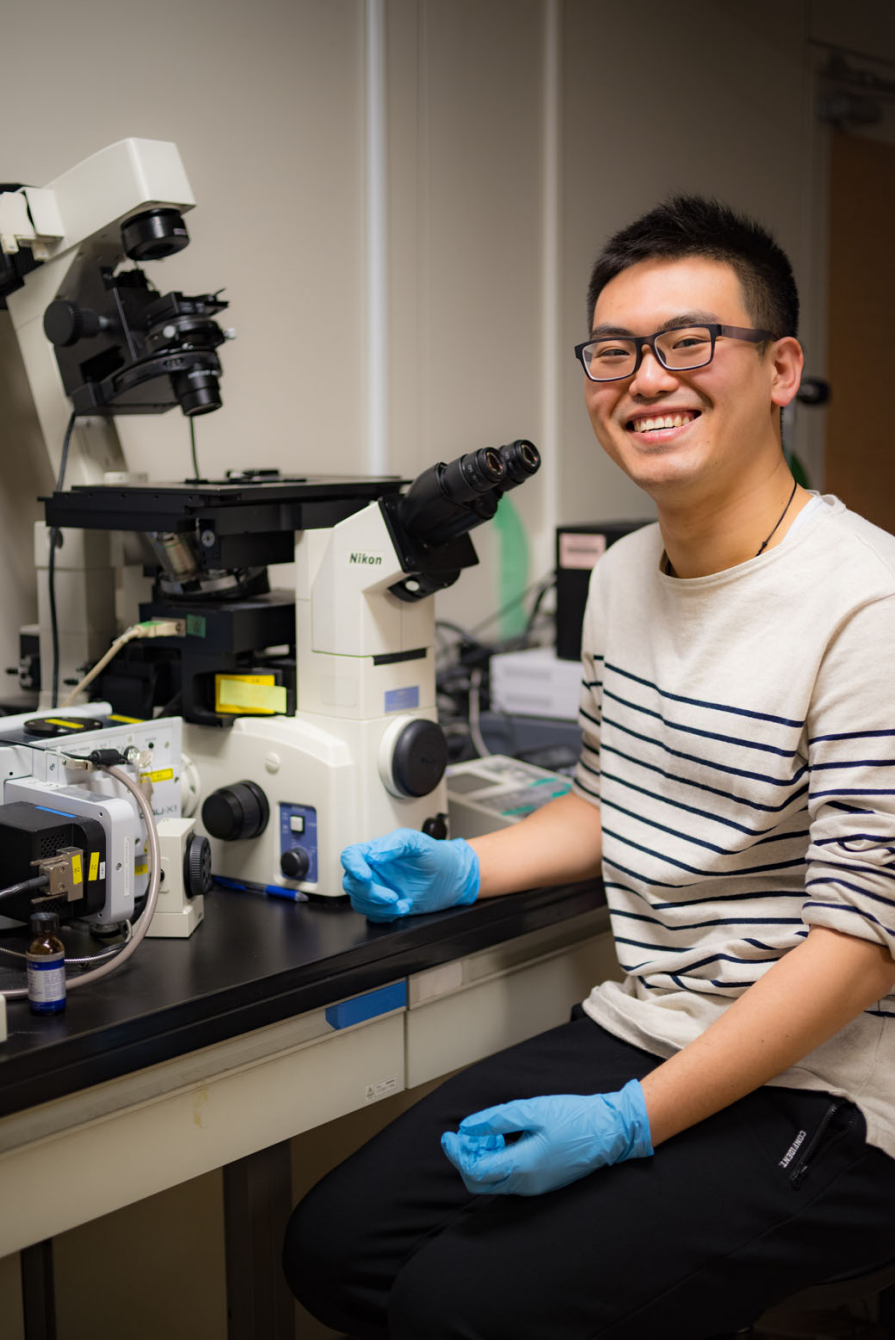
Guang Yang

**Describe your scientific journey and your current research focus**

My interest in biology began during a middle school biology course, where I was first introduced to the complex architecture of cells. I was particularly intrigued by the precise regulation of cell division within individual cells, which guarantees the proper functioning of an organism. Simultaneously, I recognised that a failure in cell division does not invariably signify the end. Errors in mitosis can induce whole-genome duplication (WGD) in human somatic cells, leading to chromosomal instability and increased lethality; it also gives cells considerable potential to modify their genomes. The cells that endure this process may become pivotal in the evolution of cancer. These insights have inspired me to explore the regulation of cell division and the long-term outcomes for WGD cells. Furthermore, the profound significance of WGD cells in tumorigenesis and cancer evolution continues to fascinate me. Currently, I am expanding my research scope to the organismal level to explore the emergence of WGD cells and their role in promoting cancer development.

**Who or what inspired you to become a scientist?**

My PhD supervisor, Dr Ryota Uehara, played a crucial role in inspiring me to become a researcher. His passion and rigor for scientific inquiry, along with his sincere expectations and encouragement for young scholars, deeply moved and motivated me. He helped me realise that the scientific research we dedicate ourselves to is noble work and showed me the importance of passing this legacy on to future generations.

In this study, through detailed lineage tracking of cells following WGD, we found that fewer than 10% of lineages survive long-term

**Figure:**
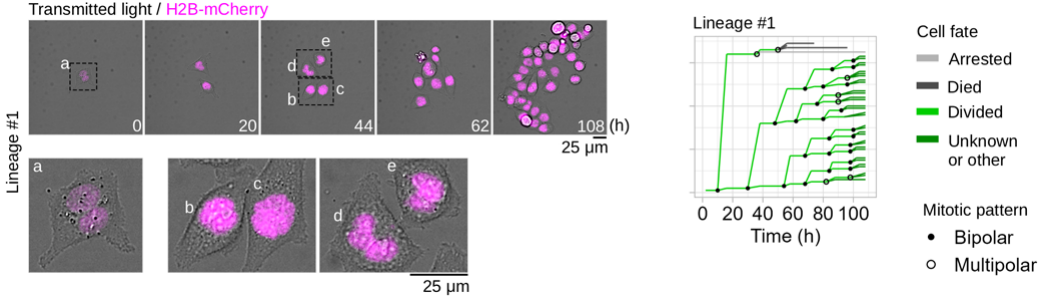
**Example of live images (left) and lineage tracing (right) of post-WGD cell.** (Left) The entire view of the lineage and magnified views of individual cells are shown in the top and bottom, respectively. The initial post-WGD cells are shown in panel a. Representative cells that experienced bipolar (b,c) or multipolar chromosome segregation (d,e) in the previous mitosis are also shown. (Right) Markers indicated different mitotic patterns. Progenies were colour-coded by cell fate.

**How would you explain the main finding of your paper?**

WGD cells arising from mitotic failure exhibit significant changes in their properties, playing vital roles in processes such as cell differentiation, senescence, and carcinogenesis. However, the specific evolutionary process by which a WGD cell population produces progeny capable of long-term proliferation remains unclear. In this study, through detailed lineage tracking of cells following WGD, we found that fewer than 10% of lineages survive long-term. This outcome depends on the timing and extent of multipolar chromosome segregation – a cell division event that causes chaotic chromosome distribution among daughter cells. Additionally, we classified the surviving WGD lineages and uncovered a unique survival strategy: cells offload the risks of multipolar chromosome segregation to a subset of progeny, enabling the remaining progeny to proliferate successfully.

**What are the potential implications of this finding for your field of research?**

In this study, we conducted continuous fluorescence microscopy imaging of newly generated WGD cells for six consecutive days, analysing more than 150 cell lineages. Because the migration and division patterns of WGD cell populations are extremely complex, tracking their lineages while minimizing phototoxicity is very challenging. This difficulty is precisely why this process had previously remained a ‘black box’ without detailed analysis, making our concrete characterization especially valuable. Through this observation, we found that although cells undergoing multipolar chromosome segregation are generally inviable, a fraction still has the potential to develop into stably proliferating cell clones. Investigating this process provides essential baseline data for understanding how WGD cell survival is determined, while also deepening our understanding of the mechanisms underlying the generation of diversity within WGD cell populations.

**What's next for you?**

While WGD cells pose a risk of chromosome instability, they also serve as normal components in certain organs. The developmental origins of these WGD cells and their functions in pathological processes remain largely unknown. I am currently initiating a new postdoctoral project utilizing mouse models, which aims to detect WGD cells at the organismal level and investigate their participation in various pathological processes. I aspire to incorporate my insights and experience in cell biology into this project, thereby achieving a more profound and comprehensive understanding of the distinct roles that WGD cells play across diverse physiological and pathological contexts.
